# ﻿Parahiraciini planthoppers with elongate head from Vietnam: a new genus and species *Pumatiraciavenosa* gen. et sp. nov. and first record of *Laohiraciaacuta* Constant, 2021 (Hemiptera, Fulgoromorpha, Issidae)

**DOI:** 10.3897/zookeys.1166.101444

**Published:** 2023-06-08

**Authors:** Jérôme Constant, Thai Hong Pham

**Affiliations:** 1 Royal Belgian Institute of Natural Sciences, O.D. Taxonomy & Phylogeny – Entomology, Vautier street 29, B-1000 Brussels, Belgium Royal Belgian Institute of Natural Sciences Brussels Belgium; 2 Mientrung Institute for Scientific Research, Vietnam National Museum of Nature, VAST, 321 Huynh Thuc Khang, Hue, Vietnam Vietnam National Museum of Nature Hue Vietnam; 3 Graduate School of Science and Technology, Vietnam Academy of Science and Technology, 18 Hoang Quoc Viet, Hanoi, Vietnam Vietnam Academy of Science and Technology Hanoi Vietnam

**Keywords:** Biodiversity, Fulgoroidea, Indochina

## Abstract

The new genus *Pumatiracia***gen. nov.** is described to accommodate a new species, *P.venosa***gen. et sp. nov.** from Pu Mat National Park in Vietnam. The new genus is placed in the subtribe Parahiraciina of the Parahiraciini. It is compared with the genera *Laohiracia* Constant, 2021, *Macrodarumoides* Che, Zhang et Wang, 2012, *Pseudochoutagus* Che, Zhang et Wang, 2011, and *Rostrolatum* Che, Zhang et Wang, 2020 with which it shares possessing an elongate head. Illustrations of habitus, details, and male genitalia are given as well as a distribution map and photographs of the habitat. *Laohiraciaacuta* Constant, 2021 is recorded for the first time from Vietnam, Pu Luong National Park; living specimens and habitat are illustrated, and the distribution map updated. The Parahiraciini fauna of Vietnam now comprises 14 species belonging to 11 genera.

## ﻿Introduction

The tribe Parahiraciini Cheng & Yang, 1991 is mostly distributed in the Oriental Region and counts 29 genera (including one fossil, *Bolbossus* Gnezdilov & Bourgoin, 2016) and 96 species (including the fossil *Bolbossusbervoetsi* (Gnezdilov & Bourgoin, 2016)) (Bourgoin 2023; Zhang et al. 2020). Recently, the tribe was divided into three subtribes, separated by features of the hindwing (Bourgoin and Wang 2020). Two subtribes contain only very few taxa: Scantiniina Bourgoin & Wang, 2020 with one genus, *Scantinius* Stål, 1866 (two species) from Sundaland and Vindilisina Bourgoin & Wang, 2020 with two monospecific genera *Nisoprincessa* Gnezdilov, 2017 and *Vindilis* Stål, 1870 from Palawan in the Philippines (Bourgoin and Wang 2020). The third subtribe Parahiraciina Cheng & Yang, 1991 is by far the most diverse with 25 genera and 91 species mostly distributed in the Oriental Region (Bourgoin 2023; Zhang et al. 2020). The rather complex taxonomic history of the tribe, which was originally described as a subfamily by Cheng and Yang (1991), was recently summarized by Bourgoin and Wang (2020). However, the higher position of the tribe was subject to some changes in the recent years (Wang et al. 2016; Bourgoin and Wang 2020; Gnezdilov et al. 2020; Gnezdilov et al. 2022). The fauna of Vietnam currently counts 12 species of Parahiraciini in nine genera; all belong to the subtribe Parahiraciina (Bourgoin 2023).

Study of the recent material of Issidae in the collection of RBINS revealed an undescribed species of Parahiraciina with an elongate head from Vietnam, which could not be placed in any of the currently existing genera, as well as the presence of the recently described *Laohiraciaacuta* Constant, 2021 in northern Vietnam

The present paper aims to describe a new genus within Parahiraciina to accommodate this new species and compare it with the other genera with an elongate head among the Parahiraciina, and provide the record of an additional genus and species for the fauna of Vietnam.

## ﻿Materials and methods

The genitalia were extracted after soaking the abdomen for some hours in a 10% solution of potassium hydroxide (KOH) at room temperature. The pygofer was separated from the abdomen and the aedeagus dissected with a needle blade for examination. The whole was then placed in glycerin for preservation in a tube attached to the pin of the corresponding specimen. Photographs of collection specimens were taken with a Leica EZ4W stereo-microscope, stacked with CombineZ software, and optimized with Adobe Photoshop software; photographs from the field were taken with an Olympus Tough 6 camera. The map was produced with SimpleMappr (Shorthouse 2010). The external morphological terminology follows O’Brien and Wilson (1985), the wing venation terminology follows Bourgoin et al. (2015) and for the male genitalia, Bourgoin and Huang (1990). The classification used follows the most recent one published by Gnezdilov et al. (2022). The metatiobiotarsal formula gives the number of spines on (side of metatibia) apex of metatibia / apex of first metatarsomere / apex of second metatarsomere.

The measurements were taken as in Constant (2004) and the following acronyms are used:

**BB** maximum breadth of the body;

**BF** maximum breadth of the frons;

**BTg** maximum breadth of the tegmen;

**BV** maximum breadth of the vertexmaximum breadth of the vertex;

**LF** length of the frons at median line;

**LT** total length (apex of head to apex of tegmina);

**LTg** length of the tegmen;

**LV** length of the vertex at median line.

Acronyms used for the collections:

**RBINS**Royal Belgian Institute of Natural Sciences, Brussels, Belgium;

**VNMN**Vietnam National Museum of Nature, Hanoi, Vietnam.

## ﻿Taxonomy

**Family Issidae Spinola, 1839**

**Subfamily Issinae Spinola, 1839**

**Tribe Parahiraciini Cheng & Yang, 1991**

### ﻿Checklist of the Parahiraciini of Vietnam

*Barduniacurvinaso* Gnezdilov, 2011

*Brevicopiusgorochovi* Gnezdilov, 2017

*Brevicopiusjianfenglingensis* (Chen, Zhang & Chang, 2014)

*Flavinaacuta* Ran & Liang, 2006

*Fortuniabyrrhoides* (Walker, 1858)

*Fortuniaviridis* (Lallemand, 1942)

*Gelastyrellalitaoensis* Yang, 1994

*Laohiraciaacuta* Constant, 2021

*Pseudochoutagusrubens* Gnezdilov & Constant, 2012

*Pumatiraciavenosa* gen. et sp. nov.

*Pusulissusphiaoacensis* Bourgoin & Wang, 2020

*Tetricodespacoensis* Vanslembrouck & Constant, 2018

*Tetricodestamdaoensis* Vanslembrouck & Constant, 2018

*Thabenafrontocolorata* Gnezdilov, 2015

**Note.** All Vietnamese Parahiraciini belong to the subtribe Parahiraciina (Bourgoin and Wang 2020; Bourgoin 2023).

### ﻿Subtribe Parahiraciina Cheng & Yang, 1991

This subtribe was defined by Bourgoin and Wang (2020) based on a combination of characters of the hind wings:

1. Hindwings bilobate, strongly notched at CuP with CuP-Pcu-A1 lobe generally wider than Sc-R-MP-CuA lobe; the two lobes almost the same length.
2. Posterior margin of hindwings not or indistinctly notched at A1 _2_.
3. A2 lobe of hindwings with anal area posterior to A1 strongly reduced, much shorter and much thinner than the anterior lobes.
4. Hindwings with Sc-R-MP-CuA and CuP-Pcu-A1 lobes covered with a set of numerous transverse veins.
5. Hindwings with CuA and CuP not merging before the anterior notch.
6. Hindwings with Pcu and A1 _1_ not merging in basal half of forewing.
7. Hindwings with A2 present, not branched, or absent. In some species, a transverse a2-a1 connecting A2 with A1 at the level of its basal branching (e.g., in *Tetricodestamdaoensis* Vanslembrouck & Constant, 2018).

#### ﻿Genus Pumatiracia gen. nov.

Taxon classificationAnimaliaHemipteraIssidae

FD28F815-3B11-568E-9415-B0C3EC6CB181

https://zoobank.org/C389D7E6-5FB1-4D47-8D64-E78E04A1FA41

##### Type species.

*Pumatiraciavenosa* gen. et sp. nov. by present designation.

##### Diagnosis.

The genus can be separated from all other Parahiraciini genera by the following combination of characters:

1. Head with vertex elongate, 1.2 × longer in median line than maximal width, projecting well beyond eyes in dorsal view, but not forming a real cephalic process;
2. Vertex and frons with a median carina;
3. Genae with strong carina under the antennae;
4. Body elongate, more than twice a long as maximum width, with side margins broadly rounded in dorsal view;
5. Metatibiae with three lateral and eight apical spines;
6. Anal tube of male dorsoventrally flattened, subtriangular in dorsal view furcate distally;
7. Posterior margin of pygofer broadly rounded;
8. Gonostyli with capitulum strongly projecting dorsad;
9. Aedeagus evenly curved in lateral view, with pair of elongate symmetrical, lateroventral processes projecting cephalad.

##### Description.

**Figure 1.**
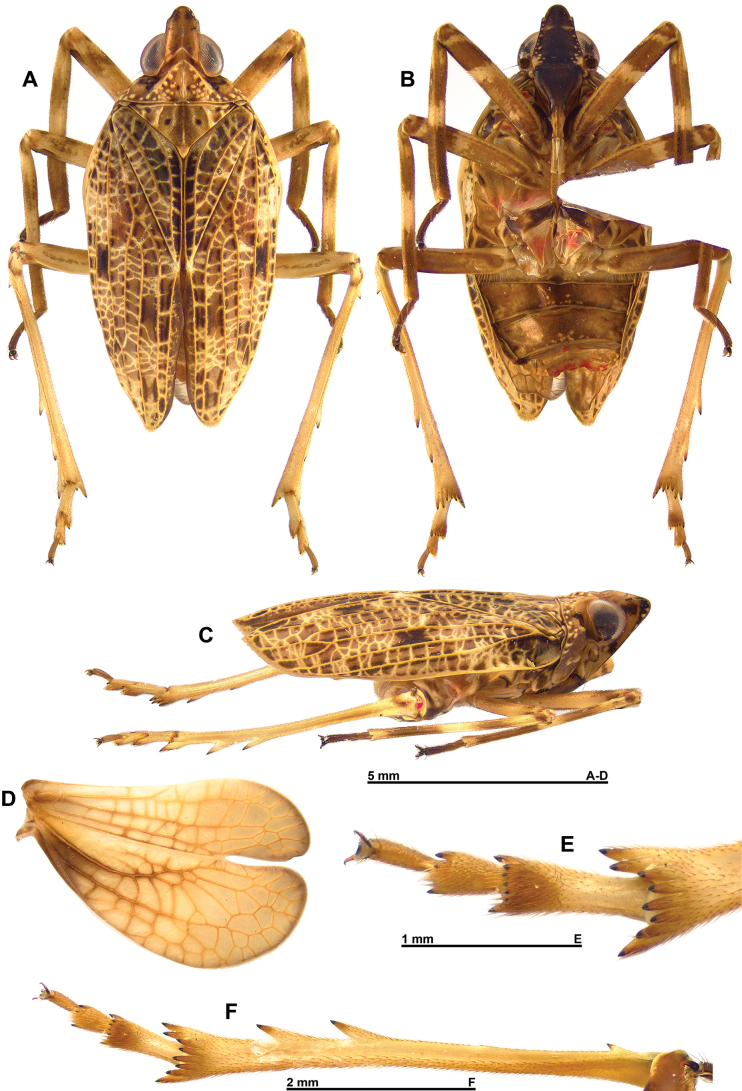
*Pumatiraciavenosa* gen. et sp. nov., holotype ♂ **A** habitus, dorsal view **B** habitus, ventral view **C** habitus, lateral view **D** right hind wing **E** metatarsus, detail, ventral view **F** metatibia and metatarsus, ventral view.

**Figure 2.**
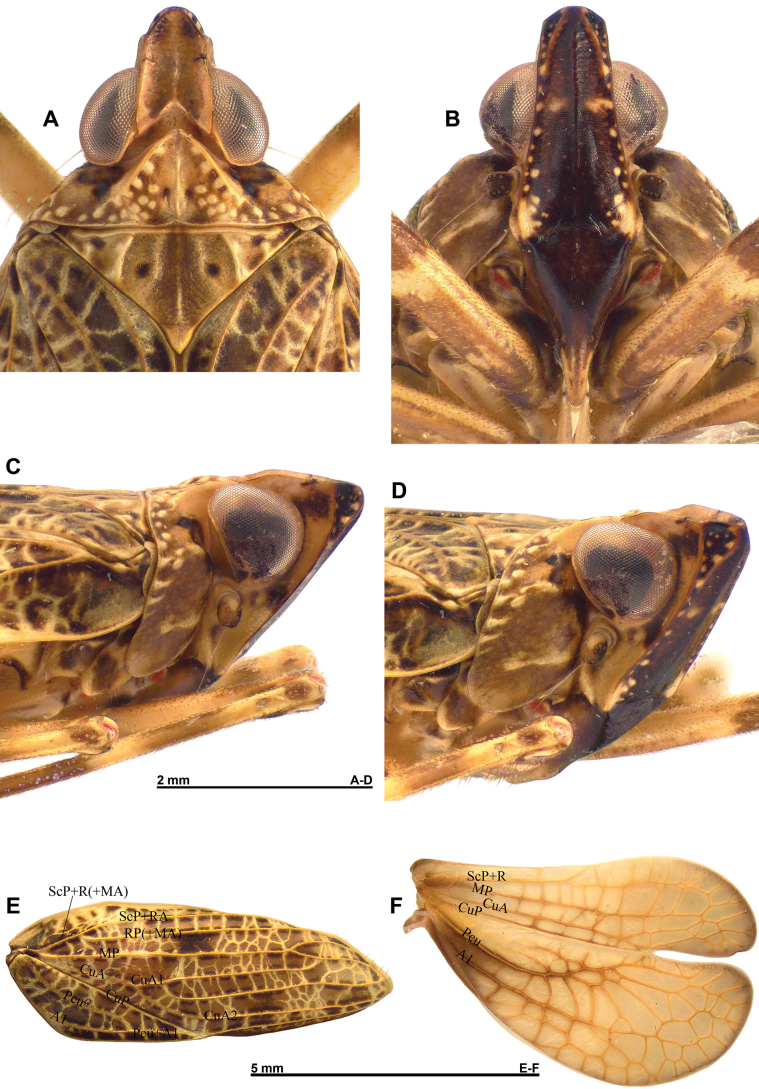
*Pumatiraciavenosa* gen. et sp. nov., holotype **A–D** detail of head and thorax **A** dorsal view **B** perpendicular view of frons **C** lateral view **D** anterolateral view **E, F** venation of wings **E** right tegmen **F** right hind wing.

***Head***: (Figs 1A–C, 2A–D) Head narrower than thorax and elongate but without forming a definite cephalic process. Vertex elongate, as long in mid-line as pronotum, ~ 1.2 × as long in mid-line as broad basally, with sides subparallel, slightly converging towards rounded apex; strong median carina not reaching apex; lateral margins carinate with carinae merging anteriorly; posterior margin of vertex angularly emarginate and carinate. Frons elongate, mostly flat with median carina, wider above fronto-clypeal suture, slightly tapering towards rounded dorsal margin; lateral margins carinate; oblique lateral carinae on distal ¼, merging dorsally with anterior carina of vertex. Genae with blunt carina running from under the antenna obliquely to fronto-clypeal suture. Clypeus flat in middle portion, moderately elongate, subtriangular with fronto-clypeal suture rounded; anteclypeus with median blunt carina. Labium elongate and narrow, reaching metacoxae, with apical segment elongate, nearly as long as penultimate. Eyes reniform (not emarginate) protruding laterally; ocelli absent. Antennae rather short with scape ring-shaped and pedicel cylindrical, slightly longer than broad.

***Thorax***: (Figs 1A, C, 2A–D) Pronotum slightly shorter than mesonotum in mid-line; anterior margin carinate in middle portion, strongly sinuate and strongly, roundly protruding anteriorly between eyes; posterior margin nearly truncate; median carina obsolete anteriorly with impressed point on each side; blunt tubercles along anterior margin and irregularly on disc and sides; paranotal lobes (lateral view) broad, with tubercles, sometimes turning into short, blunt, longitudinal carinae along posterior margin, with blunt carina behind level of antenna, and with posteroventral angle rounded. Mesonotum subtriangular with obsolete median carina and transverse anterior carina; disc smooth with additional, more or less undulate, longitudinal carina; weak, blunt slightly rounded longitudinal carina on each side of disc; some blunt tubercles in lateral fields of mesonotum. Tegulae moderately developed.

Tegmina: (Figs 1A–C, 2E) Tegmina subcoriaceous with longitudinal veins elevated and with a dense reticulum of veinlets, elongate with sides broadly rounded, ~ 2.7 × longer than broad, convex. Apex narrowly rounded. Postclaval margin weakly rounded on distal half and slightly notched at apex of clavus. Clavus closed, reaching ca. mid-length of tegmen.

Venation: ScP+R moderately developed, forking into subparallel ScP+RA and RP; MP forking at ~ ¾ of tegmen length; CuA forked slightly after half of clavus length; CuA_1+2_ forked close to its base; CuA_1_, CuA_2_ and CuA_3_ subparallel, with CuA_1_ merging distally with MP_2_; Pcu fused with A1 at ¾ of clavus length; Pcu+A1 fused with CuP slightly before apex of clavus.

Hind wings: (Figs 1 D, 2 F) Broader than tegmina and deeply bilobed, strongly notched at CuP; costal margin rather weakly sinuate; CuP-Pcu-A1 lobe ~ 1/3 wider than Sc-R-MP-CuA lobe, the two lobes almost the same length; both lobes rounded apically; postclaval margin broadly rounded; A2 lobe reduced and narrow, with A2 vein obsolete.

Venation: main veins present; ScP+R, MP and CuA running more or less parallel, slightly diverging towards apex, with numerous cross-veins; Pcu strongly curved around basal third of wing towards CuP but not reaching the latter; A1 curved, more or less parallel to postclaval margin; CuP-Pcu-A1 lobe with numerous cross-veins.

Legs: (Fig. 1A–C, E, F) Strongly elongate and slender. Pro- and mesofemora and protibiae slightly flattened dorsoventrally. Tibiae longer than corresponding femora. Metatibiae with one lateral tooth near base, two lateral teeth placed on distal half and eight apical teeth. Tarsi elongate; first metatarsomere elongate and slender, with a strong spine at each side and a row of six smaller spines in between ventrally along posterior margin; second metatarsomere short with one tooth at each side. Metatibiotarsal formula: (3) 8 / 8 / 2.

**Figure 3.**
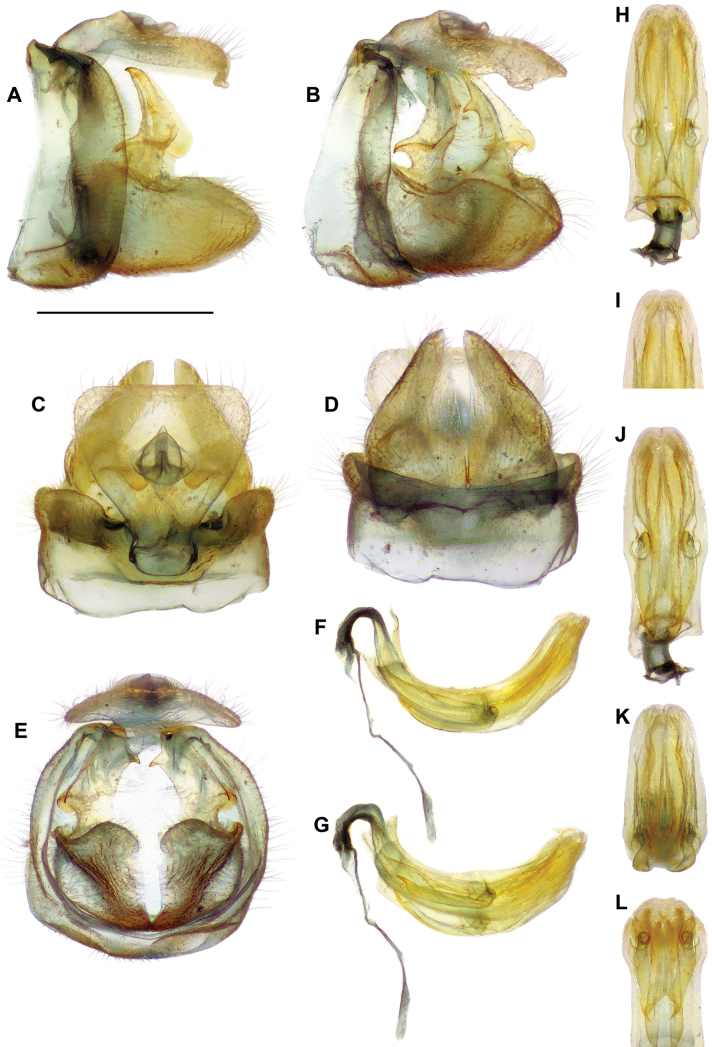
*Pumatiraciavenosa* gen. et sp. nov., holotype, terminalia ♂ **A–E** pygofer, gonostyli and anal tube **A** lateral view **B** posterolateral view **C** dorsal view **D** ventral view **E** posterior view **F–L** aedeagus, phallobase and connective **F** left lateral view **G** left lateroventral view **H** dorsal view **I** apical portion, anterodorsal view **J** ventral view **K** posteroventral view **L** anteroventral view.

***Terminalia*** ♂: (Fig. 3) Pygofer higher than long in lateral view, with anterior margin weakly concave and posterior margin broadly rounded. Gonostyli (in lateral view) elongate and broad, projecting posteriorly, with capitulum strongly developed dorsad, with lateral laminate projection and with moderately developed neck with outer margin strongly concave in posterior view; in ventral view, gonostyli abruptly narrowing after basal ¼. Anal tube dorsoventrally flattened, broadly subtriangular. Aedeagus moderately curved dorsad (in lateral view), with symmetrical pair of elongate medioventral processes directed cephalad. Connective elongate.

##### Etymology.

The new genus name is formed by the combination of *Pu Mat*, referring to the National Park where the new genus was found, and *hiracia*, which is the same ending as in *Parahiracia* Ouchi, 1940 (synonymized by Gnezdilov et al. (2004) under *Fortunia* Distant, 1909), the type genus of the tribe Parahiraciini and a reminder of the placement of the new genus in this group. The gender is feminine.

##### Distribution.

Vietnam.

#### ﻿ Pumatiracia venosa

Taxon classificationAnimaliaHemipteraIssidae

gen. et sp. nov.

8D01042B-9A2F-5711-ABF4-07083AD98F39

https://zoobank.org/866560E0-E51F-46D9-9489-6DFF942615C4

**Figure 4.**
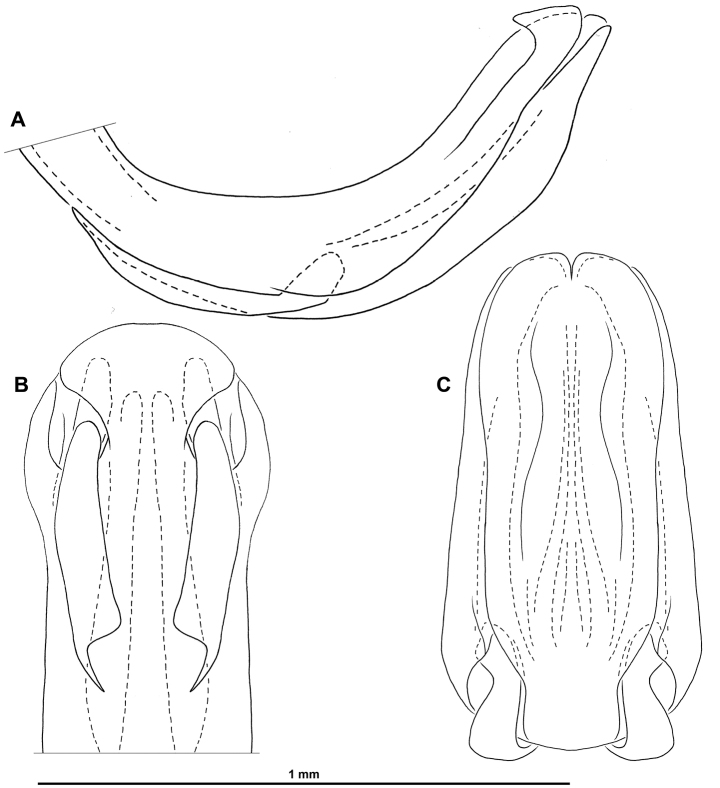
*Pumatiraciavenosa* gen. et sp. nov., holotype ♂, aedeagus **A** left lateral view **B** anteroventral view **C** posteroventral view.

**Figure 5.**
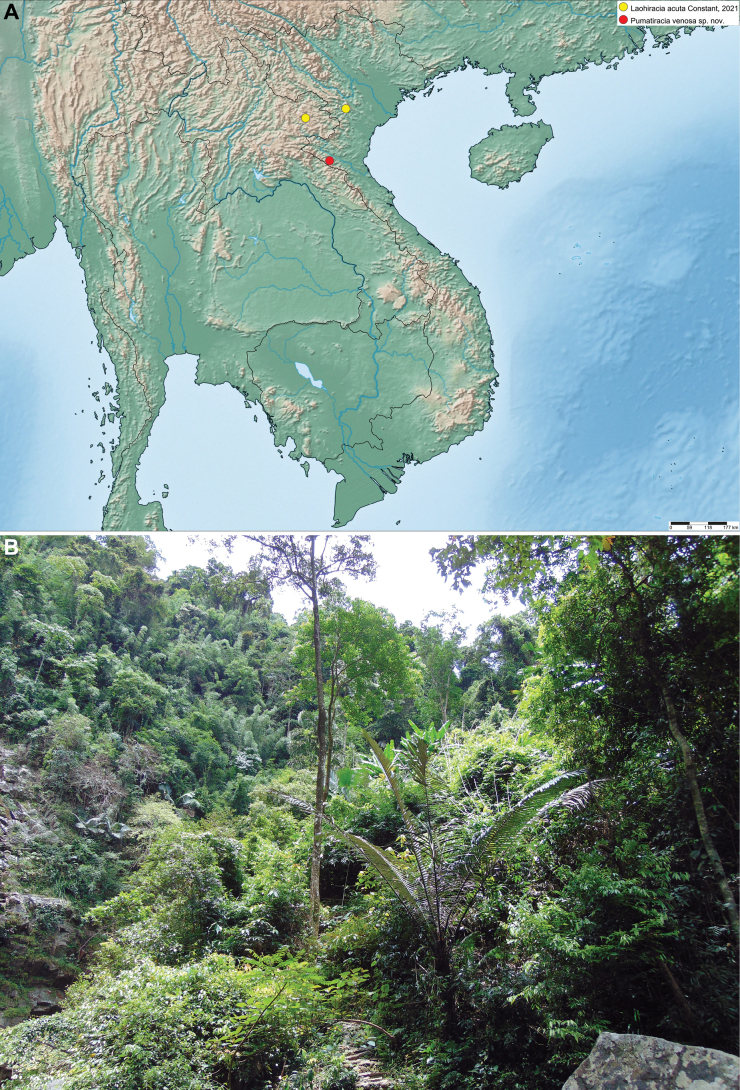
**A** distribution map of *Pumatiraciavenosa* gen. et sp. nov. and *Laohiraciaacuta* Constant, 2021 **B** habitat of *Pumatiraciavenosa* gen. et sp. nov. in Vietnam, Pu Mat National Park, 6 July 2017.

Figures 1 , 2 , 3 , 4 , 5

##### Type material.

***Holotype* ♂, Vietnam** • Nghe An Province, Pu Mat National Park; 18°59'N, 104°40'E; 4–9.VII.2017; [sweeping]; GTI project; leg. J. Constant & J. Bresseel; I.G.: 33.498; RBINS.

##### Diagnosis.

Only species in the genus. The shape of the vertex, 1.2 ×longer than broad, and characters of the male terminalia, are probably relevant diagnostic characters to recognize the species, e.g., the gonostyli strongly projecting posteriorly in lateral view and with a dorsal process strongly elongate dorsad, the widely subtriangular anal tube (in dorsal aspect) and the shape of the ventral processes of the aedeagus.

##### Description.

***Measurements and ratios***: LT: ♂ (*n* = 1): 8.86 mm. Lt/BB = 2.12; LTg/BTg = 2.74; LW/BW = 1.50; LV/BV = 1.2; LF/BF = 1.86.

***Head***: (Fig. 2A–D) Narrower than thorax and elongate, with ~ 1/2 of vertex length surpassing eyes. Vertex yellowish, strongly concave, ~ 1.2 × as long in mid-line as broad basally, with sides slightly converging towards rounded apex, small black marking on laterodistal angles; strong median carina not reaching apex, yellowish; lateral, anterior, and posterior margins carinate; posterior margin angularly concave. Frons mostly shiny black, with rather large yellowish marking at angles of fronto-clypeal suture and series of small yellowish points along lateral margins, a larger transverse yellowish marking at level of mid-length of eye on each side of median carina; above latter markings, slightly transversely wrinkled; oblique lateral carinae on distal ¼, brown. Posterior side of head yellowish. Genae yellowish with black-brown marking between eye and anterodorsal angle and between anteroventral angle of eye and lateral margin of frons, the two markings linked by a fine black line along anterior margin of eye; blunt carina running from under the antenna obliquely to fronto-clypeal suture, with small brown marking under antenna. Clypeus shiny black, flat in middle portion, moderately elongate, subtriangular with fronto-clypeal suture rounded; anteclypeus mostly yellowish with median blunt carina. Labium yellowish, elongate, and narrow, reaching metacoxae, with apical segment elongate, nearly as long as penultimate. Eyes reniform (not emarginate) protruding laterally; ocelli absent. Antennae yellowish with anterior portion dark brown, rather short with scape ring-shaped and pedicel cylindrical, slightly longer than broad.

***Thorax***: (Fig. 2A–D) Pronotum variegated yellowish brown with large black-brown marking on anterolateral portion (behind eye); 0.76 × as long as mesonotum in mid-line; anterior margin carinate in middle portion, strongly sinuate and strongly, roundly protruding anteriorly between eyes, with carinae directed obliquely posteriorly, not reaching hind margin of pronotum; posterior margin nearly straight; median carina obsolete anteriorly with impressed point on each side; rather large blunt, pale yellowish tubercles along anterior margin’s carina and irregularly on disc and sides; paranotal lobes (lateral view) broad, brown with pale yellowish tubercles, sometimes turning into short, blunt, longitudinal carinae along posterior margin, with pale yellowish blunt carina behind level of antenna, and with posteroventral angle rounded. Mesonotum variegated yellowish brown with two black points on disc and one additional black point more laterally along anterior carina; subtriangular with obsolete median carina and nearly complete transverse anterior carina; disc smooth, slightly excavate, with additional, more or less undulate, longitudinal carina; weak, blunt slightly rounded longitudinal carina on each side of disc; some blunt, pale yellowish tubercles in lateral fields of mesonotum. Tegulae yellowish brown.

Tegmina: (Figs 1A, C, 2E) Variegated yellowish brown with darker area in clavus between Pcu-Pcu+A1 and margin, a black subrectangular marking at ca. mid-length of tegmina between veins RP and MP; subcoriaceous with longitudinal veins pale yellow, elevated and with a dense reticulum of pale yellow veinlets; shape elongate and convex with sides broadly rounded, ~ 2.7 × longer than broad; narrowly rounded apically. Postclaval margin weakly rounded on distal half and slightly notched at apex of clavus. Clavus closed, reaching ca. mid-length of tegmen.

Venation: (Fig. 2E) ScP+R moderately developed, forking into subparallel ScP+RA and RP; MP forking at ~ ¾ of tegmen length; CuA forked slightly after half of clavus length; CuA_1+2_ forked close to its base; CuA_1_, CuA_2_ and CuA_3_ subparallel, with CuA_1_ merging distally with MP_2_; Pcu fused with A1 at ¾ of clavus length; Pcu+A1 fused with CuP slightly before apex of clavus.

Hind wings: (Figs 1D, 2F) Yellow brown with venation slightly darker to brown (base of ScP+R+MP and most of Pcu and A1), apical margin of both lobes narrowly infuscate; wing broader than tegmen and deeply bilobed at CuP; costal margin rather weakly sinuate; CuP-Pcu-A1 lobe ~ 1/3 wider than ScP-R-MP-CuA lobe, the two lobes almost the same length; both lobes rounded apically; postclaval margin broadly rounded; A2 lobe brown, reduced and narrow, with A2 vein obsolete.

Venation: main veins present; ScP+R, MP and CuA running more or less parallel, slightly diverging towards apex, with numerous cross-veins; Pcu strongly curved around basal third of wing towards CuP but not reaching the latter: A1 curved, more or less parallel to postclaval margin; CuP-Pcu-A1 lobe with numerous cross-veins.

Legs: (Fig. 1A–C, E, F) Coxae and trochanters yellowish with dark brown marking. Pro- and mesofemora dorsally yellowish with weakly marked brown band in distal half, ventrally mostly brown, darker towards apex and with two oblique yellowish bands, one just after half-length and one narrower slightly before apex; elongate, slender and slightly flattened dorsoventrally. Pro- and mesotibiae brown with yellowish ring basally and wider yellowish ring at ca. mid-length, darker ventrally; tibiae slightly flattened dorsoventrally and narrower and longer than corresponding femora. Pro- and mesotarsi elongate and brown. Metatibiae yellowish dorsally and brown ventrally, with one lateral tooth near base, two lateral teeth placed on distal half and eight apical teeth, all teeth dark brown distally. Tarsi yellowish, brown ventrodistally, elongate; first metatarsomere elongate and slender, with a strong spine at each side and a row of six smaller spines in between ventrally along posterior margin; second metatarsomere short with one tooth at each side, all spines black-brown apically. Metatibiotarsal formula: (3) 8 / 8 / 2.

***Abdomen***: (Fig. 1B) Brown.

***Terminalia*** ♂: (Figs 3, 4) Pygofer ~ 2.8 × higher than long in lateral view, with anterior margin weakly concave and posterior margin broadly rounded. Gonostyli (in lateral view) ~ 1.6 × as long as high (without dorsal capitulum), projecting posteriorly and strongly concave; capitulum strongly developed dorsad, ca. as high as pygofer in lateral view, with distal portion anteroposteriorly flattened, with apical hook and one tooth in middle of inner margin, with lateral laminate projection bearing one anterolateral and one posterolateral tooth, with moderately developed neck with outer margin strongly concave in posterior view; in ventral view gonostyli ~ 1.9 × as long as wide, abruptly narrowing after basal ¼, with margin obliquely straight on most length. Aedeagus moderately curved dorsad (in lateral view), ~ 2.5 × as long as wide in dorsal view, with apical small triangular process dorsally on each side, reflexed cephalad; symmetrical pair of elongate medioventral processes directed cephalad, reaching anterior margin of periandrium and strongly emarginate at inner margin apically; dorsal lobe of periandrium more or less parallel-sided, slightly tapering on distal portion and roundly truncate apically; ventral lobe of periandrium slightly widening in distal half then tapering towards apex and apically rounded with median notch. Connective elongate with small tectiductus (damaged in examined specimen). Anal tube broadly subtriangular, slightly wider than long, dorsoventrally flattened with short ventrally reflexed projection in middle of posterior margin, with posterolateral angles rounded; anal opening at ca. mid-length.

##### Etymology.

The species epithet *venosus* is a Latin adjective meaning ‘relating to the veins’ and refers to the raised veins of the tegmina in this species.

##### Biology.

The species was found in subtropical forest (Fig. 5B) belonging to the Northern Indochina subtropical forests ecoregion, at medium altitude (900–1,600 m).

##### Distribution.

Vietnam, Nghe An Province, Pu Mat National Park (Fig. 5A).

##### Note.

The color of living specimens is probably brighter green than in collection specimens, as shown for example for *Laohiracia* Constant, 2021 (compare Fig. 5A–C to Constant 2021: fig. 1 A, E).

#### ﻿Genus Laohiracia

Taxon classificationAnimaliaHemipteraIssidae

Constant, 2021

C2B15A24-126D-5F72-9EEA-3283DB3645D8

Laohiracia Constant, 2021: 5. Type species: Laohiraciaacuta Constant, 2021 by original designation.

#### ﻿ Laohiracia acuta

Taxon classificationAnimaliaHemipteraIssidae

Constant, 2021

A8FE9F62-33D5-59DD-BAD0-31C740D9F66A

Figs 5A , 6

Laohiracia acuta Constant, 2021: 8, figs 1–4.

##### Note.

The species was described from Laos, Hua Phan province, Mt Phu Pane, 900–1600 m. It is here recorded from Vietnam for the first time.

##### Material examined.

**Vietnam** • 2♂♂, 3♀♀; Thanh Hoa Province, Pu Luong National Park; 20°27'48"N, 105°07'38"E; 5–10 Aug. 2022; 1700 m; [sweeping]; GTI project; leg. J. Constant, J. Bresseel & L. Semeraro; I.G.: 34.518; RBINS • 2♂♂, 2♀♀; same collection data as preceding; VNMN.

##### Biology.

**Figure 6.**
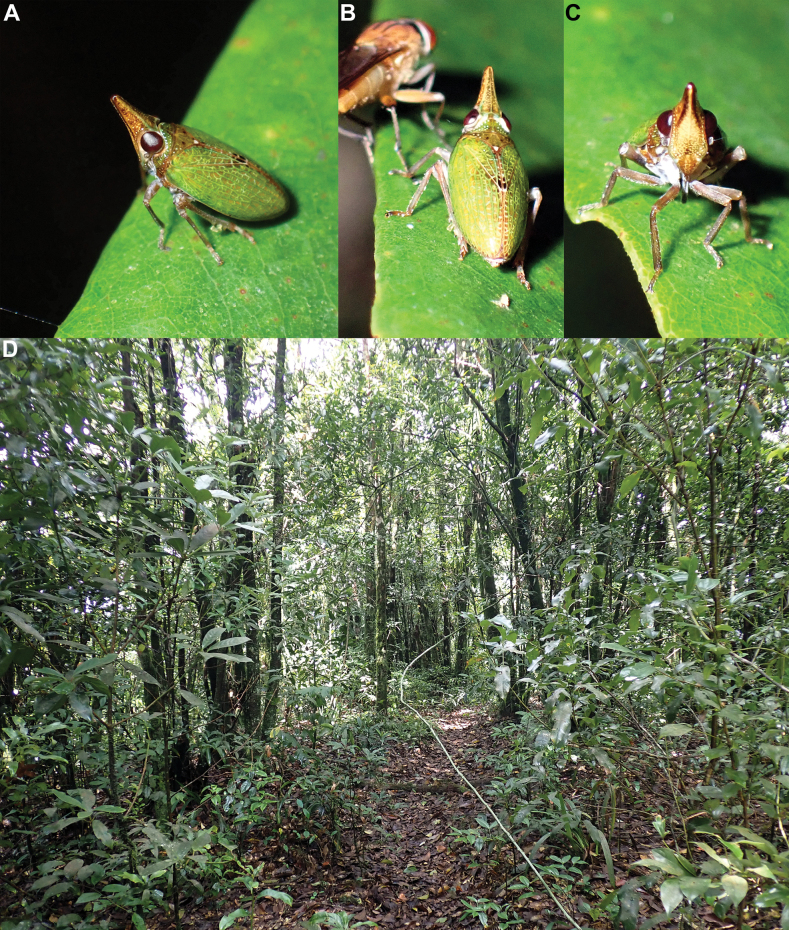
*Laohiraciaacuta* Constant, 2021 in Vietnam, Pu Luong National Park, summit of Mount Pu Luong, 1700 m, 8 August 2022 **A–C** live specimen **D** habitat.

The species was found in mountain forest (Fig. 6D) belonging to the northern Indochina subtropical forests ecoregion, at the summit of Mount Pu Luong (1700 m). The specimens were collected by sweeping and visual screening of the lower vegetation and bushes up to 2 m high; they were sitting on the leaves (Fig. 6A–C). The trees growing on the summit are considerably shorter (usually < 10 m in height) in size than those growing in the well-preserved forests on the slopes and down the mountain (trees > 30 m). This also provides a habitat with more light than in the rest of the forest. The species seemed to be restricted to the summit as extensive sampling along the slopes of Mount Pu Luong and at lower altitude in Pu Luong National Park, did not provide any additional specimen, although other Issidae species were collected (pers. obs.). The host plant is unknown.

##### Distribution.

(Fig. 5A) Laos: Hua Phan Province; Vietnam: Thanh Hoa Province (new country record).

## ﻿Discussion

The new genus *Pumatiracia* gen. nov. is one of the few Parahiraciina genera with an elongate head (vertex). Within the subtribe, only four other genera share the condition of an elongate head: *Laohiracia* Constant, 2021, *Macrodarumoides* Che, Zhang & Wang, 2012, *Pseudochoutagus* Che, Zhang & Wang, 2011 and *Rostrolatum* Che, Zhang & Wang, 2020 (Che et al. 2011, 2012; Zhang et al. 2020; Constant 2021).The genera *Pseudochoutagus* and *Laohiracia* can be separated by possessing a strongly developed cephalic process (see also Constant 2021 for illustrations of both genera), with the vertex more than twice as long in mid-line, as wide basally (~ 1.2 × in *Pumatiracia*). The genus *Rostrolatum* can be separated from *Pumatiracia* by possessing a triangular vertex (vertex more or less parallel-sided in *Pumatiracia*), and by its frons strongly concave in lateral view (frons oblique, straight in *Pumatiracia*) (see Zhang et al. 2020: 337, fig. 133, pl. XXV J–L). The genus *Macrodarumoides* can be separated from *Pumatiracia* by possessing a triangular vertex (vertex more or less parallel-sided in *Pumatiracia*), the shape of its tegmina, much wider at basal 1/3 (tegmina with sides broadly rounded in *Pumatiracia*) and 11 apical spines of metatibia (8 in *Pumatiracia*).

The new genus also shows some similarities with the genus *Flavina* Stål, 1861, of which the type species, *F.granulata* Stål, 1861 was illustrated in Gnezdilov and Wilson (2007) and other species in Ran and Liang (2006), Zhang et al. (2010) and Chen et al. (2014); additional undescribed species of *Flavina* from Vietnam were also examined (pers. obs.). However, *Pumatiracia* gen. nov. can be separated from *Flavina* by its distinctly elongate vertex (~ 1.2 × as long in mid-line, as wide basally versus 0.8–1.0 × in *Flavina*), the upper margin of the frons (in perpendicular view of frons) convex as opposed to concave in *Flavina* and especially by the strong vertical carina on the genae under the antennae (Fig. 2C, D), always absent in the genus *Flavina*.

Together with the taxa added in this paper, the Vietnamese fauna of Parahiraciini counts 14 species in 11 genera. Although Vietnamese Parahiraciini are highly endemic at species level, nine of the 11 genera are shared with China and one with Laos, leaving one endemic genus for the country, *Pumatiracia* gen. nov.; four genera (*Bardunia* Stål, 1863, *Flavina* Stål, 1861, *Fortunia* Distant, 1909 and *Thabena* Stål, 1866) even show a wider distribution in the Oriental Region with *Thabena* being recorded as far West as in Indian Ocean islands (Gnezdilov 2009; Bourgoin 2023); the latter genus was even reported from Europe (France, Portugal, and Spain) by Melichar (1906) but this data was never confirmed. However, many new taxa still need to be described in the future which will provide a more accurate view of the fauna of Vietnam (pers. obs.).

## Supplementary Material

XML Treatment for Pumatiracia

XML Treatment for Pumatiracia venosa

XML Treatment for Laohiracia

XML Treatment for Laohiracia acuta

## ﻿Additional information

### Author ORCIDs

Jérôme Constant https://orcid.org/0000-0003-0254-0863

Thai Hong Pham https://orcid.org/0000-0002-4763-3679

### Data availability

All of the data that support the findings of this study are available in the main text or Supplementary Information.
